# O-Band 4 × 1 Combiner Based on Silicon MMI Cascaded Tree Configuration

**DOI:** 10.3390/mi17010031

**Published:** 2025-12-26

**Authors:** Saveli Shaul Smolanski, Dror Malka

**Affiliations:** Faculty of Engineering, Holon Institute of Technology (HIT), Holon 5810201, Israel

**Keywords:** multimode interference, optical power combiner, beam propagation method, O-band

## Abstract

High-speed silicon (Si) photonic transmitters operating in the O-band require higher on-chip optical power to support advanced modulation formats and ever-increasing line rates. A straightforward approach is to operate laser diodes at higher output power or employ more specialized sources, but this raises cost and exacerbates nonlinear effects such as self-phase modulation, two-photon absorption, and free-carrier generation in high-index-contrast Si waveguides. This paper proposes a low-cost 4 × 1 tree-cascade multimode interference (MMI) power combiner on a Si-on-insulator platform at 1310 nm wavelength that enables coherent power scaling while remaining fully compatible with standard commercial O-band lasers. The device employs adiabatic tapers and low-loss S-bends to ensure uniform field evolution, suppress local field enhancement, and mitigate nonlinear phase accumulation. The optimized layout occupies a compact footprint of 12 µm × 772 µm and achieves a simulated normalized power transmission of 0.975 with an insertion loss of 0.1 dB. Spectral analysis shows a 3 dB bandwidth of 15.8 nm around 1310 nm, across the O-band operating window. Thermal analysis shows that wavelength drift associated with ±50 °C temperature variation remains within the device bandwidth, ensuring stable operation under realistic laser self-heating and environmental changes. Owing to its broadband response, fabrication tolerance, and compatibility with off-the-shelf laser diodes, the proposed combiner is a promising building block for O-band transmitters and photonic neural-network architectures based on cascaded splitter and combiner meshes, while preserving linear transmission and enabling dense, large-scale photonic integration.

## 1. Introduction

Optical power combiners are fundamental components in modern photonic integrated circuits, enabling compact, low-loss signal routing and power distribution for data communication and sensing systems [1,2,3,4,5]. In short-reach optical interconnects, particularly in the O-band around 1310 nm, the continuous drive toward higher line rates (800 Gb/s, 1.6 Tb/s, and beyond) has intensified the need for highly efficient on-chip power-combining schemes [6,7,8]. In such transmitters, modulators, passive routing, filtering elements, and packaging interfaces introduce several decibels of optical loss, thereby significantly tightening the link budget. As a result, higher optical power is required at the transmitter output than can typically be delivered by a single commercial O-band laser operated at standard drive conditions, which motivates the development of on-chip power-scaling techniques.

A straightforward approach is to drive laser diodes at higher output power or to employ specialized higher-power laser modules. However, these solutions increase system cost and reduce energy efficiency, and they may also exacerbate nonlinear effects such as self-phase modulation, two-photon absorption, and free-carrier generation in high-index-contrast silicon (Si) waveguides [9,10]. Under such conditions, these nonlinearities can degrade signal integrity and limit the linear dynamic range of advanced modulation formats. An alternative strategy is to coherently or quasi-coherently combine multiple moderate-power channels from standard commercial lasers using low-loss on-chip power combiners, thereby increasing the available optical power while preserving linearity and cost-effectiveness.

Among various combining mechanisms, multimode interference (MMI) couplers have emerged as reliable and fabrication-tolerant solutions due to their self-imaging property, simple geometry, and broadband operation [11,12]. MMI theory enables precise control of optical field replication inside a multimode region, supporting a wide range of power-splitting and power-combining configurations [13,14,15]. Continued development of MMI-based devices has led to implementations based on conventional Si waveguides, slot-waveguide structures, and hybrid material platforms [16,17,18,19]. These designs demonstrate stable performance, improved polarization tolerance, and enhanced modal control, thereby establishing MMIs as versatile building blocks for integrated photonics.

Material platforms play a critical role in determining the optical and thermal performance of MMI devices. Si, Si-nitride, Si-gallium nitride, and alumina have each been explored for wavelength-division multiplexing and power-combining applications, owing in particular to their low absorption in the O-band spectral range [20,21]. Slot-waveguide configurations, in which the optical field is strongly confined in a low-index region, can further enhance power efficiency and reduce device footprint [22]. Fabrication-tolerant layouts and hybrid dielectric structures improve the achievable insertion loss and bandwidth uniformity across the O-band [23,24,25]. Nevertheless, for multi-input combiners on high-index-contrast Si platforms, the strong field confinement that enables compactness may increase sensitivity to sidewall roughness, wavelength variation, and nonlinear effects at elevated optical intensities [26,27].

To alleviate these limitations, recent works have proposed cascaded and multi-stage MMI layouts, often based on slot-waveguide geometries, to distribute interference more evenly and suppress parasitic reflections [28]. Although slot-waveguide MMIs can offer strong field confinement, they typically require tighter lithographic control and more complex process steps. In contrast, the present work employs a buried Si waveguide MMI realized on a Si-on-insulator (SOI) platform, which is fully compatible with conventional complementary-metal-oxide-semiconductor (CMOS) fabrication flows and thereby simplifies practical implementation. The proposed tree-cascade 4 × 1 MMI power combiner is designed and numerically optimized for O-band operation, targeting low insertion loss, broad spectral robustness, and high fabrication tolerance.

By combining four moderate-power inputs from commercial O-band laser sources, the proposed architecture increases the available on-chip optical power without resorting to expensive high-power laser modules and may help reduce nonlinear penalties in the combining region. The design targets low insertion loss, broad spectral stability with a 3 dB bandwidth of 15.8 nm, and high fabrication and thermal tolerance, thereby providing a suitable solution for next-generation high-speed O-band transmitters. In addition, the compact 4 × 1 combiner can serve as a fundamental building block in photonic neural-network architectures that rely on cascaded networks of splitters, combiners, and active amplifying or weighting elements for implementing tunable synaptic weights and large-scale matrix operations [29].

## 2. Structural Design and Theoretical Analysis

**Figure 1:**
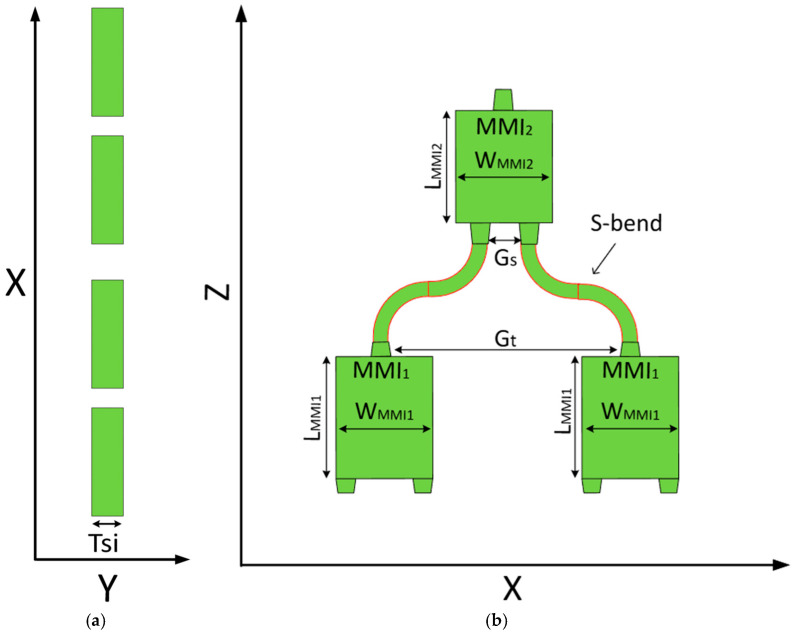
(**a**) Top-view (x–y) cross-section of the four Si input waveguides, embedded in a SiO_2_ cladding. (**b**) Side-view (x–z) schematic of the tree-cascade MMI structure, including the tapered inputs, S-bend connections, and upper combining stage.

The proposed 4 × 1 power combiner is implemented on a SOI platform, which provides high refractive-index contrast and enables strong optical confinement throughout the device. This material configuration supports compact routing and efficient control of modal evolution. Figure 1a illustrates the x-y cross-section of the proposed combiner. In this configuration, the high-index regions (shown in green) correspond to Si (n = 3.48) formed on a 220 nm-thick SOI platform marked as T_Si_. The surrounding cladding (shown in white) is silicon dioxide (SiO_2_) with a refractive index of 1.44.

Figure 1b presents the x-z plane cross-section at Y = 0, showing the four input tapers, the two MMI_1_ couplers, an output taper following each MMI_1_, the pair of S-bend waveguides connecting MMI_1_ to MMI_2_, the two input tapers leading into MMI_2_, the MMI_2_ coupler, and the output taper. The input tapers in MMI_1_ extend over an L_taper_ of 15 μm, and gradually expand from 0.5 μm to 0.8 μm, while the output transition has the same length and contracts from 0.8 μm back to 0.5 μm. A second taper length marked as L_taper2_ of 22.5 μm is also implemented with the same width transition for improved mode matching in the upper combining stage. The gap (G_t_) between the output tapers of each MMI_1_ is 7.2 μm.

Both MMI_1_ constitute the first combining stage, merging the four input channels into two intermediate paths. These two paths are routed upward using S-bend waveguides with their widths matched to the 0.5 μm taper output. The S-bend length is 242.5 μm, while having an offset of 2.6 μm, and the center-to-center spacing (G_s_) at the S-bend outputs is set to be 2 μm.

Finally, MMI_2_ performs the final combining step, producing a single output channel at the top of the device. The overall device length is 772 μm.

The operation principle of the MMI device relies on the self-imaging effect, in which the input optical field is periodically reproduced along the propagation axis of the multimode region. The key parameters governing this effect are the effective width, beat length, and MMI length, given by the following equations:

The beat length of the MMI combiner is expressed as [30]:

(1) $$L_{\pi }=\frac{4n_{eff}{W_{e}}^{2}}{3\lambda },$$

where the operating wavelength is denoted by **λ**, the effective refractive index of the guided mode is represented by *n_eff_*, and the effective width of the multimode interference region is denoted by *W_e_*.

The effective width depends both on the physical width and the polarization state. For TE polarization, it is defined as [30]:

(2) $$W_{e}=W_{MMI}+\frac{\lambda }{\pi }\left(\frac{n_{clad}}{\sqrt{{n_{eff}}^{2}-{n_{clad}}^{2}}}\right),$$

where *n_clad_* is the refractive index of the silica cladding.

The length of the MMI region that supports self-imaging is then given by [30]:

(3) $$L_{\mathrm{MMI}}=\frac{3{\mathrm{pL}}_{\pi }}{4N},$$

where p is a positive integer, and N is the number of input sources. In this work, N = 2 for each individual MMI and p = 1 were selected to obtain a compact device length.

The optical bandwidth and fabrication tolerance of MMI couplers are known to depend strongly on these geometrical parameters, as small width or length variations can significantly alter the self-imaging condition.

The insertion loss (IL) of the combiner is described by:

(4) $$\mathrm{IL}\left[\mathrm{dB}\right]=-10\log_{10}\left(\frac{\mathrm{Pout}}{N\;\mathrm{Pin}}\right),$$

where P_out_ is the output optical power, and P_in_ is the power of a single input source (thus N P_in_ represents the total launched power for equal-amplitude inputs).

For simulation purposes, a multi-input excitation (N = 4) was applied in the beam propagation method (BPM) using its multi-objective optimization tool (MOST) analyses, while the full device operates as a 4 × 1 combiner under coherent illumination.

In addition to the parameters defining the MMI region, the performance of the combiner strongly depends on the geometry of the tapered transitions and S-bend sections. To reduce bending-related losses, the geometry of the S-bend sections was selected with particular care. Prior studies indicate that an offset of 5 µm is a suitable choice for Si waveguides [31]. For a given offset O and S-bend length L, the corresponding bend radius R can be obtained from the geometric relationship:

(5) $$R=\frac{L^{2}+O^{2}}{4O},$$

In our design, substituting L and O into Equation (5) yields a bend radius of 5.65 mm. This relatively large radius is well above the typical minimum bend radius used in SOI waveguides and thus keeps bend-induced radiation loss in the S-bend sections negligible.

Unlike conventional MMI combiners, where inputs are symmetrically aligned, the proposed layout routes signals through curved geometries to support multi-layer interconnectivity. The tree-cascade MMI configuration further enhances robustness by enabling gradual mode evolution and reducing sensitivity to fabrication errors. These design features allow the combiner to serve as a high-efficiency component suitable for scalable photonic integration.

The analytical self-imaging relations provide an initial estimate for the MMI length under idealized assumptions. Because the proposed SOI layout includes access tapers and routing sections that affect the modal excitation and effective propagation constants, the final MMI dimensions were determined by full-device BPM optimization.

## 3. Simulation Results

Comprehensive numerical simulations were carried out using the BPM module in RSoft CAD (v2024.09) to validate the optical performance of the proposed 4 × 1 MMI power combiner. The analysis considers both the modal characteristics and the field propagation along the entire device, including the S-bend transitions, tapered input sections, and the tree-cascade MMI regions. The key design parameters, namely the S-bend geometry, MMI width, and MMI length, were systematically varied in order to maximize transmission efficiency and minimize IL over the O-band. The results presented in this section include the geometrical optimization of the MMI regions, optical field propagation maps, and the spectral response of the combiner.

**Figure 2:**
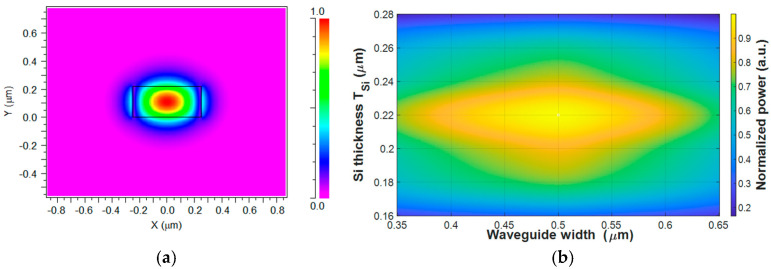
(**a**) Simulated fundamental mode profile in the Si core (x–y plane) for the nominal input waveguide geometry (500 nm × 220 nm). (**b**) Two-dimensional tolerance map of the normalized combined output power as a function of input waveguide width and T_Si_ around the nominal design point.

To accurately model light propagation within the structure, the fundamental guided mode of the input buried Si waveguide was obtained using the Mode Solver in RSoft. The input waveguide consists of a Si core with dimensions of 500 nm (width) × 220 nm (thickness), embedded in a SiO_2_ cladding. Figure 2a shows the transverse electric (TE) field component (E_x_) of the fundamental TE_0_ mode for the Si core, with an effective refractive index of 2.73. It can be seen that strong optical confinement is achieved within the Si core, with the field exhibiting evanescent decay into the surrounding cladding. This field distribution represents the steady-state modal solution of the Si core buried waveguide. The mode was normalized to unit power and exported as a field file for use as the input excitation in the subsequent BPM simulations. Figure 2b presents the optimization and tolerance analysis of the input Si waveguide as a function of width and silicon thickness. The optimal dimensions are 500 nm × 220 nm, where the normalized power reaches its maximum value. A robust tolerance window is observed around this point, where simultaneous deviations of approximately ±20 nm in both width and thickness maintain >90% of the normalized power. This indicates that the proposed design is relatively tolerant to realistic fabrication variations.

**Figure 3:**
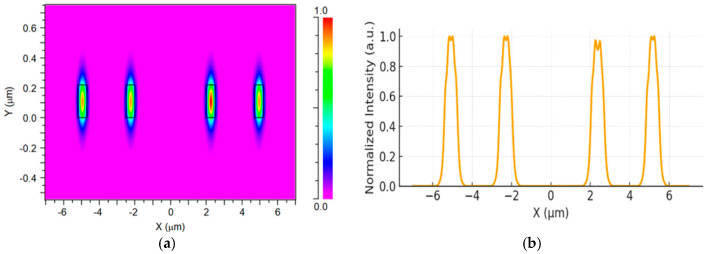
(**a**) TE_0_ field distribution (x–y plane) of the four Si input taper waveguides at 1310 nm wavelength. (**b**) E_x_ intensity profile along the x-axis (Y = 0).

Figure 3a shows the transverse electric field distribution (E_x_) of the fundamental TE_0_ mode across the four input Si taper waveguides. The simulation exhibits four well-confined field regions, each localized within its corresponding Si core. The effective refractive index of the guided mode is calculated to be 2.73, thereby confirming single-mode operation in each input waveguide for the selected geometrical and material parameters. The strong light confinement within each Si core, together with the negligible field overlap between adjacent channels, indicates that inter-channel coupling and crosstalk at the input section are minimal. To further illustrate the mode uniformity, Figure 3b presents a one-dimensional line profile extracted along the central horizontal axis (Y = 0) of the same field map. The profile exhibits four distinct and symmetric intensity peaks associated with the four input waveguides. Each peak reaches a normalized amplitude close to 1, whereas the regions between neighboring peaks drop nearly to 0, forming two pairs of peaks separated by a low-intensity valley. This symmetry in the intensity distribution verifies that all four input channels are well balanced in optical power and that the simulated field distribution is spatially periodic and uniform across the input array.

The balanced modal intensity is crucial for maintaining equal optical weighting when the fields propagate into the MMI region. Such uniform inputs guarantee that constructive and destructive interference in the MMI section will occur predictably, thereby ensuring high-efficiency optical power combination and minimal phase imbalance at the output.

To achieve high-efficiency optical power combining, the lengths and widths of both MMI sections were optimized using a parametric sweep with the MOST module in RSoft. The optimization was performed with BPM by launching the simulated fundamental TE mode at the inputs and modeling the complete cascaded structure (including access tapers and interconnecting S-bends). Therefore, the reported optimal MMI lengths correspond to the integrated device performance, rather than to an isolated, idealized slab-MMI estimate. The discrepancy from the analytical self-imaging length is expected because the analytical model relies on simplified assumptions (e.g., uniform multimode region and ideal excitation) and does not fully capture modal dispersion, effective-index variations, and transition-induced changes in modal excitation in high-index-contrast SOI implementations. The optimization process aimed to identify the structural dimensions that provide maximum output power and minimal IL within the O-band.

**Figure 4:**
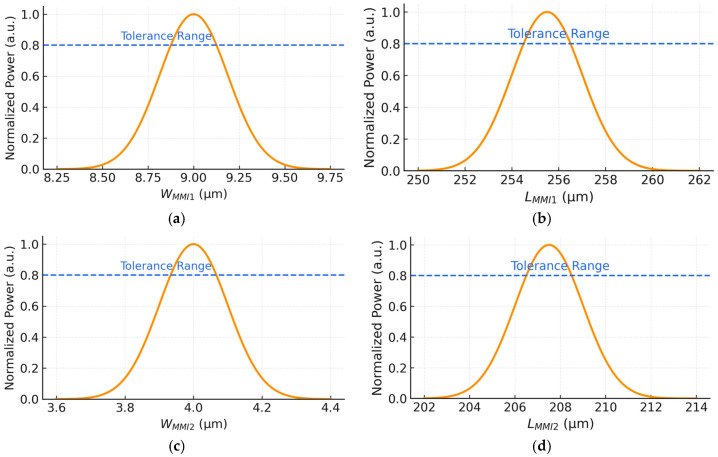
Normalized output power as a function of the MMI geometrical parameters. (**a**) W_MMI1_. (**b**) L_MMI1_. (**c**) W_MMI2_. (**d**) L_MMI2_.

Figure 4a presents the normalized output power as a function of W_MMI1_. The curve reaches its optimized value at 9 µm, where constructive interference between the guided modes is strongest. Importantly, the output remains above 80% of the peak across a tolerance window of ±0.15 µm, showing that the selected value lies at the center of a robust, fabrication-tolerant region.

Similarly, Figure 4b shows the length optimization of L_MMI1_, with a simulated optimum of 255.5 µm. Using the analytical expressions for effective width (Equation (2)) and beat length (Equation (1)), MMI_1_ yields an effective width of 9.25 µm, a beat length of 237.74 µm, and a calculated self-imaging length of roughly 267.45 µm from Equation (3). The difference between these analytical predictions and the simulated optimum follows the expected trend for SOI MMIs, where BPM optimization refines the idealized self-imaging estimate. The device maintains high performance within a tolerance of roughly ±6 µm, confirming that the chosen simulated length is stable under standard lithographic variations.

The MMI_2_ stage, responsible for further field consolidation, was optimized in the same manner. As shown in Figure 4c, W_MMI2_ exhibits maximum transmission at 4 µm, with a tolerance band of ±0.1 µm. Despite its smaller geometry, the optimal point lies well inside the allowable tolerance range, preserving strong modal overlap and low IL.

Lastly, Figure 4d illustrates the optimization of L_MMI2_. The optimal performance is obtained at 207.5 µm, again with a tolerance of ±6 µm. For reference, the analytical expressions for effective width and beat length (Equations (1) and (2)) yield an effective width of 4.25 µm and a beat length near 50.18 µm for this stage, and the corresponding self-imaging estimate from Equation (3) gives a calculated length of roughly 18.81 µm. As expected, these closed-form values differ from the BPM-optimized result, which accounts for full vectorial field evolution and taper-induced modal reshaping. The selected simulated value, therefore, ensures proper self-imaging and stable performance across typical dimensional variations, enabling reliable two-to-one combining into the output waveguide.

Overall, these results confirm that both MMI sections exhibit stable optical behavior over moderate fabrication variations. Maintaining normalized power above 0.8 across the tolerance ranges demonstrates that the device is highly robust and manufacturing-friendly for integration within photonic systems.

**Figure 5:**
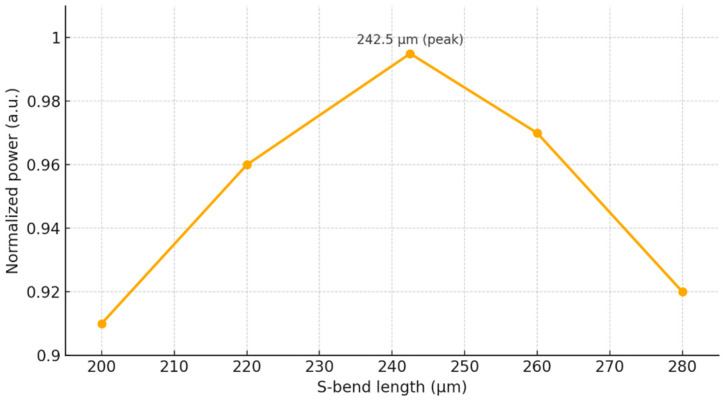
Normalized output power versus S-bend length.

To support low-loss routing between the lower and upper combining stages, the interconnecting waveguides were implemented using smooth S-bend transitions. Their geometry was evaluated using the MOST optimization tool, and the resulting normalized transmission is presented in Figure 5. The simulated trend follows the expected behavior. Very short bends suffer from increased radiation loss, and as the length increases, the transmission rises, reaching a maximum at an S-bend length of 242.5 µm. For longer bends, the transmitted power gradually decreases due to additional propagation loss. From this figure, the normalized power remains above 0.9 for S-bend lengths between approximately 200 µm and 280 µm, corresponding to a tolerance of about ±40 µm around the optimum value. This wide tolerance window indicates that the S-bend is highly robust to dimensional variations and can be reliably fabricated using standard CMOS-compatible processes.

**Figure 6:**
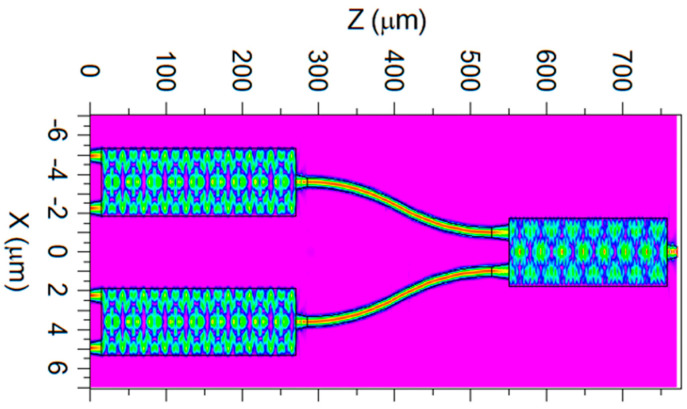
Simulated field propagation (x–z plane) of the optimized 4 × 1 power combiner.

Figure 6 shows the simulated optical field evolution in the designed 4 × 1 power combiner at 1310 nm under simultaneous excitation of all four input ports with equal amplitudes and in-phase operation (zero relative phase difference). The field evolution confirms stable confinement within the Si core across both MMI stages and along the interconnecting S-bend transitions. The optical energy follows the intended combining path without noticeable radiation into the silica cladding, indicating that the chosen geometry supports low-loss propagation. The output region exhibits the expected interference pattern associated with the final combining stage, with a well-defined mode emerging at the device exit. Overall, the field distribution demonstrates that the device maintains high transmission and robust modal behavior across the full 772 µm propagation length.

**Figure 7:**
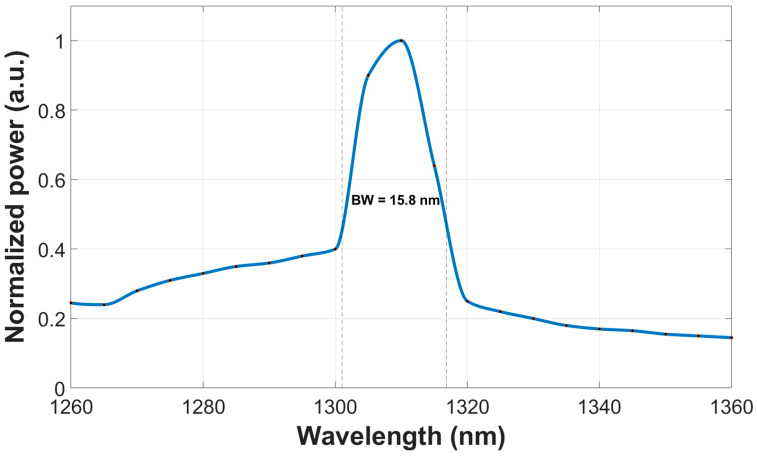
Spectral response of the combiner, showing peak transmission at 1310 nm and a well-defined 3 dB bandwidth across the O-band.

The spectral response of the proposed combiner over the O-band (1260–1360 nm) was evaluated using BPM simulations, in which the fundamental mode at each wavelength was first obtained from the mode solver and then post-processed using a MATLAB (R2024a) script, as shown in Figure 7. The device exhibits high transmission at the operating wavelength of 1310 nm. Applying the 3 dB criterion (normalized power of 0.5), the resulting optical bandwidth is 15.8 nm, extending from approximately 1301 nm to 1317 nm. These results demonstrate that the tree-cascade MMI combiner maintains high transmission efficiency within a 15.8 nm window around 1310 nm and can therefore operate effectively in the O-band region commonly employed in Si photonic communication systems.

To assess thermal sensitivity, the thermo-optic coefficient of Si was taken into account, leading to an effective wavelength shift of approximately 0.1 nm/°C. A temperature excursion of 50 °C, therefore, produces a spectral shift of about ±5 nm around the nominal peak at 1310 nm. From a simulation perspective, the 3 dB bandwidth is mainly constrained by the wavelength dependence of the MMI mechanism, since deviations from the design wavelength introduce phase mismatch among the guided modes and degrade the self-imaging condition. From Figure 7, the normalized transmission at the shifted wavelengths (1305 nm and 1315 nm) remains between about 0.64 and 0.90, corresponding to a worst-case thermally induced excess loss of 1.9 dB with respect to the peak value. As shown in Figure 7, the device exhibits a 3 dB bandwidth of 15.8 nm, extending from approximately 1301 nm to 1317 nm, so this ±5 nm drift remains well within the high-efficiency operating window of the combiner. This bandwidth is primarily determined by the wavelength dependence of the MMI mechanism, as efficient power combining relies on maintaining the relative phase relationships among the guided modes within the multimode region. Deviations from the design wavelength lead to progressive phase mismatch, which degrades the self-imaging condition and defines the 3 dB operating window. Consequently, the output transmission and self-imaging behavior are only weakly affected by moderate temperature variations, indicating that the proposed design is thermally stable and suitable for reliable operation in dense Si photonic integration platforms.

**Figure 8:**
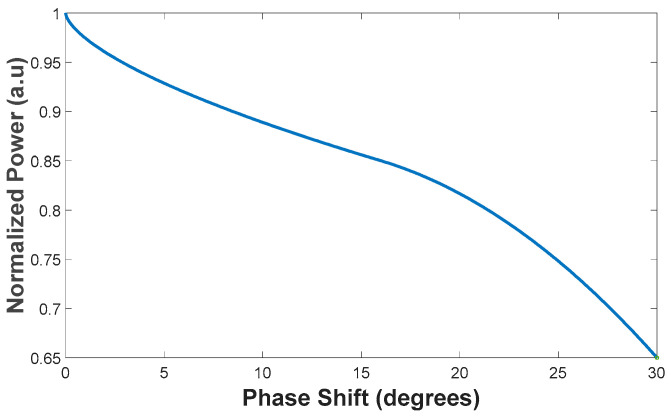
Normalized output power of the proposed combiner as a function of the relative phase shift between the non-coherent input sources.

A further consideration from a practical perspective is that the lasers may operate as mutually non-coherent sources. To quantify the associated performance penalty, simulations were performed in which independent phase offsets were applied to each of the four input sources. Figure 8 presents the normalized output power as a function of the relative (overall) phase mismatch among the four non-coherent inputs. The proposed combiner maintains high efficiency, achieving ≥90% combining efficiency for phase deviations between 0° and 8.49°. However, larger phase mismatches lead to a noticeable reduction in combining performance; for example, at a 30° phase shift, the combining efficiency decreases to 65.1%. To mitigate this sensitivity, an active phase-control stage should be integrated at the input waveguide section to align the phases of the four sources. This functionality can be implemented using on-chip phase shifters, for instance, based on a doped Si rib waveguide heater [32], a titanium nitride (TiN) microheater [33], or a PIN-diode phase-shifting structure [34].

To benchmark the proposed 4 × 1 combiner, a comparative study was carried out against previously reported MMI-based power combiners implemented on InP/InGaAsP and SOI platforms. The main design metrics, namely the footprint, number of input lasers, IL, and operating bandwidth, are summarized in Table 1. The comparison indicates that the proposed tree-cascade SOI combiner simultaneously achieves ultra-low IL and a compact layout while supporting four input lasers in the O-band.

In particular, the simulated IL is on the order of 0.1 dB, which is significantly lower than the multi-dB losses reported for earlier MMI combiners. Although the device footprint of 12 µm × 772 µm is only moderately larger in area than the most compact SOI structure reported for two inputs, the proposed combiner accommodates four inputs and substantially reduces the propagation length compared with other four-input designs. Moreover, it is the only device in the comparison for which a quantified 3 dB bandwidth is provided. The measured bandwidth of 15.8 nm around 1310 nm shows that, despite the inherent wavelength sensitivity of the self-imaging mechanism, careful optimization of the MMI dimensions and S-bend transitions yields a broad operating window. This combination of ultra-low IL, compact footprint, and verified wide bandwidth makes the proposed combiner highly suitable for integration in dense silicon photonic circuits, where power efficiency and spectral robustness are critical. The insertion loss values reported in Table 1 are presented as representative values taken from the respective references, derived from either numerical simulations or experimental measurements, depending on the original work.

## 4. Conclusions

A 4 × 1 SOI power combiner based on a tree-cascade MMI architecture has been designed, optimized, and numerically evaluated for O-band operation at 1310 nm. The structure employs buried Si waveguides with adiabatic tapers and low-loss S-bend transitions in order to maintain controlled modal evolution and suppress radiation loss throughout the combining network. BPM simulations were used to determine the optimal geometrical parameters of the two MMI stages and the interconnecting S-bends, resulting in efficient self-imaging and a compact overall footprint of 12 µm × 772 µm.

At the nominal operating wavelength of 1310 nm, the device exhibits a normalized transmitted power of 0.975, which corresponds to an insertion loss of approximately 0.1 dB. Parametric optimization of the MMI widths and lengths shows that the normalized power remains above 0.8 within realistic width and length deviations, and the S-bend analysis indicates that normalized power above 0.9 is maintained for a wide range of bend lengths around the optimum value. These results confirm that the proposed geometry is highly tolerant to fabrication-induced dimensional variations and is compatible with standard CMOS processing.

The spectral response over the O-band was obtained by combining BPM simulations with a wavelength-by-wavelength mode solver and post-processing in MATLAB. Applying the 3 dB criterion with a normalized power level of 0.5, the combiner achieves a 3 dB bandwidth of 15.8 nm, extending from approximately 1301 nm to 1317 nm around the 1310 nm peak. This bandwidth is significant for a self-imaging-based device and demonstrates that careful optimization of the multimode region and taper transitions can overcome the intrinsic wavelength sensitivity of MMI structures. In addition, thermal analysis that incorporates the thermo-optic coefficient of silicon shows that a temperature variation of ±50 °C produces a wavelength shift of about ±5 nm, which remains well inside the obtained 3 dB bandwidth. The associated excess loss remains below 2 dB, indicating that the combiner maintains stable performance under realistic self-heating and environmental conditions.

A comparison with previously reported MMI-based power combiners implemented on InP/InGaAsP and SOI platforms shows that the proposed device combines several key advantages. It supports four input channels while achieving an IL that is significantly lower than the multi-dB values typically reported for similar functions. At the same time, the footprint is competitive with existing SOI implementations, and the required propagation length is notably shorter than that of other four-input designs. Furthermore, among the devices considered, the present work is the only one that reports a quantified 3 dB bandwidth, which provides a clear indication of the usable operating window for system-level design.

In summary, the 4 × 1 tree-cascade MMI combiner presented in this work offers ultra-low insertion loss, a compact footprint, wide optical bandwidth, and robust tolerance to both fabrication deviations and thermal drift. These characteristics make it a strong candidate for integration in high-speed O-band transmitters, coherent power-combining front ends, and scalable silicon photonic circuits, including photonic neural network architectures that rely on cascaded meshes of splitters, combiners, and active weighting elements. Future work may address experimental realization, polarization-diverse operation, and extension of the architecture to larger input counts and other wavelength bands.

## Figures and Tables

**Table 1:** Comparison between the proposed power combiner and previously reported MMI-based designs.

MMI Power Combiner Type	Footprint (µm^2^) (Width × Length)	Number of Lasers	IL (dB)	Bandwidth (nm)	Year
2-Stage combiner based on InP/InGaAsP [35]	5 × 1700	4	4.03	N/A	2013
MMI based on InP/InGaAsP waveguide [36]	6 × 1720	2	0.77	N/A	2015
MMI based on SOI strip waveguide [37]	5 × 142.186	2	2	N/A	2015
Tree-Cascade MMI 4 × 1 combiner (SOI)	12 × 772	4	0.1	15.8	In this work

## Data Availability

The original contributions presented in this study are included in the article. Further inquiries can be directed to the corresponding author.
